# eIF5A-Independent Role of DHPS in p21^CIP1^ and Cell Fate Regulation

**DOI:** 10.3390/ijms222413187

**Published:** 2021-12-07

**Authors:** Andrew E. Becker, Pui-Kei Wu, Jong-In Park

**Affiliations:** Department of Biochemistry, Medical College of Wisconsin, Milwaukee, WI 53226, USA; abecker@mcw.edu (A.E.B.); pkwu@mcw.edu (P.-K.W.)

**Keywords:** deoxyhypusine synthase, eukaryotic initiation factor 5A, p21CIP1, cell death, growth arrest, BRAF, ERK1/2

## Abstract

Deoxyhypusine synthase (DHPS) catalyzes the first step of hypusination of the elongation translation factor 5A (eIF5A), and these two proteins have an exclusive enzyme–substrate relationship. Here we demonstrate that DHPS has a role independent of eIF5A hypusination in A375 and SK-MEL-28 human melanoma cells, in which the extracellular signal regulated kinase 1/2 (ERK1/2) pathway is deregulated. We found that RNA interference of DHPS induces G0/G1 cell cycle arrest in association with increased p21^CIP1^ expression in these cells whereas eIF5A knockdown induces cell death without increasing p21^CIP1^ expression. Interestingly, p21^CIP1^ knockdown switched DHPS knockdown-induced growth arrest to cell death in these cells, suggesting a specific relation between DHPS and p21^CIP1^ in determining cell fate. Surprisingly, ectopic expression of DHPS-K329R mutant that cannot hypusinate eIF5A abrogated DHPS knockdown-induced p21^CIP1^ expression in these cells, suggesting a non-canonical role of DHPS underlying the contrasting effects of DHPS and eIF5A knockdowns. We also show that DHPS knockdown induces p21^CIP1^ expression in these cells by increasing *CDKN1A* transcription through TP53 and SP1 in an ERK1/2-dependent manner. These data suggest that DHPS has a role independent of its ability to hypusinate eIF5A in cells, which appears to be important for regulating p21^CIP1^ expression and cell fate.

## 1. Introduction

Deoxyhypusine synthase (DHPS) is the only known enzyme that catalyzes the first step of hypusination of the elongation translation factor 5A (eIF5A), which is the only DHPS substrate known to date [1,2]. Given this exclusive enzyme–substrate relationship, it is generally expected that DHPS and eIF5A mediate overlapping contexts of cellular processes, and although DHPS mRNA has been shown to be a natural antisense transcript for the scaffold protein MORG1 [3], no mutually independent function of DHPS and eIF5A has been reported at protein levels. Hypusination is a highly specific post-translational modification that has been detected with only eIF5A [4,5,6]. eIF5A hypusination is mediated by two step enzyme reactions that sequentially occur. First, DHPS conjugates the aminobutyl moiety of spermidine to a specific lysine residue of eIF5A to produce deoxyhypusine-eIF5A [7,8,9]. Second, deoxyhypusine hydroxylase (DOHH) reduces deoxyhypusine-eIF5A to irreversibly produce hypusine-eIF5A [10,11]. Cells express two functionally redundant highly homologous eIF5A isoforms, eIF5A1 and eIF5A2 [12]. eIF5A1 is the major isoform ubiquitously expressed in almost any tissue type, whereas eIF5A2 expression is limited to specific tissue or tumor types [13,14]. Hypusination enables eIF5A to promote translational elongation of the polypeptide chains stalling due to disruptive sequences such as polyproline-motifs and sequentially charged residues [15,16,17] as well as translational termination on a global scale [15,18,19]. As determined in yeast *Saccharomyces cerevisiae* and cells of higher organisms, eIF5A hypusination is a fundamental process for eukaryotic cell viability [20]. Expression of DHPS, DOHH, and eIF5A is often upregulated in different tumors [21], and better understanding the role of the hypusination pathway is important.

p21^CIP1/WAF^ is a cyclin-dependent kinase inhibitor (CDKI) that can mediate G0/G1 cell cycle arrest by inhibiting CDK2, CDK4, and CDK6, and subsequently preventing retinoblastoma protein (Rb) phosphorylation, E2F1 release from Rb, and entry into the S-phase of the cell cycle [22]. p21^CIP1^ has a role as a tumor suppressor along with other CDKIs such as p16^INK4A^ and p27^KIP1^ in various tumor biological contexts [23]. Particularly, p21^CIP1^ has been identified as a key mediator of growth arrest induced upon sustained activation of the Raf/MEK/extracellular-signal regulated kinase (ERK) pathway in different cell types [24]. Interestingly, although mainly known for its role to mediate cell cycle arrest in the G0/G1 phase [25], p21^CIP1^ is also known to suppress cell death [23]. The Raf/MEK/ERK pathway is a mitogenic pathway and its deregulated signaling is detected in a wide variety of cancers [26]. Therefore, it is important to elucidate the mechanisms by which the pathway regulates p21^CIP1^ expression and how p21^CIP1^ expression is suppressed in tumor cells that exhibit aberrant MEK/ERK activity.

A previous study proposed a role for DHPS in p21^CIP1^ regulation using the specific DHPS inhibitor GC7 [27]. However, it was never reported whether the DHPS-eIF5A cascade has any role in p21^CIP1^ regulation in the context of Raf/MEK/ERK signaling. We therefore sought to determine whether the DHPS-eIF5A cascade has any role in p21^CIP1^ regulation and cell fate decision in Raf/MEK/ERK-activated tumor cells. In this study, we demonstrate in the *BRAF^V600E^* melanoma cells A375 and SK-MEL-28 that DHPS knockdown induces growth arrest associated with p21^CIP1^ upregulation whereas eIF5A knockdown induces cell death, and that p21^CIP1^ knockdown can switch the growth arrest induced by DHPS knockdown to cell death. Subsequently, we show that DHPS can negatively regulate p21^CIP1^ expression independently of its eIF5A hypusinating activity, identifying a previously unknown non-canonical function of DHPS.

## 2. Results

### 2.1. DHPS Depletion Increases p21^CIP1^ Levels and Induces G0/G1 Phase Cell Cycle Arrest in A375 and SK-MEL-28 Cells

**Figure 1:**
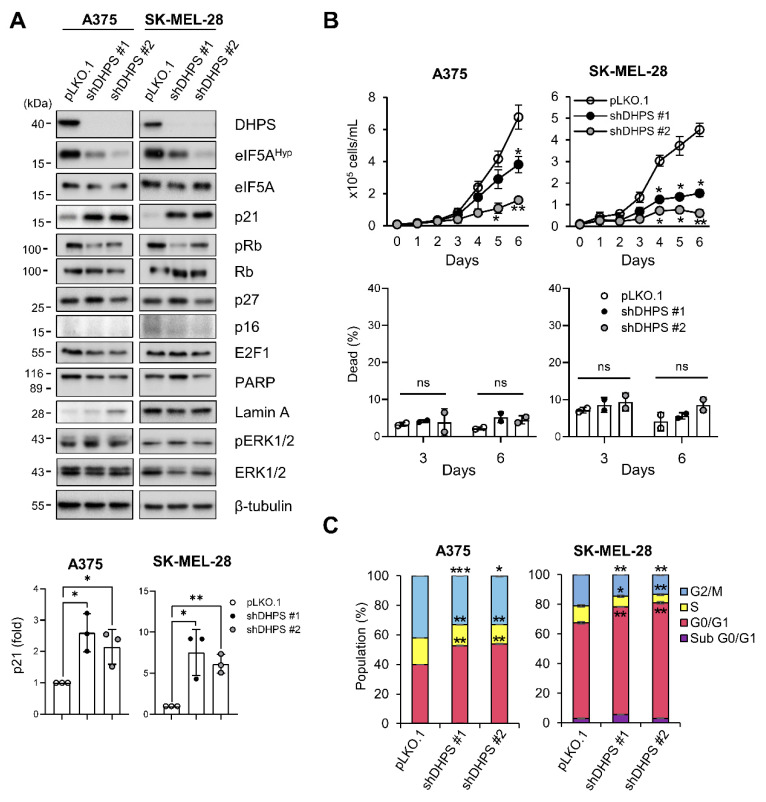
DHPS knockdown increases p21^CIP1^ levels and induces growth arrest without causing cell death in A375 andSK-MEL-28 cells. (**A**) Western blots of total lysates of cells infected for 5 days with pLKO.1 viruses expressing shRNA targeting different regions of *DHPS* mRNA (shDHPS #1 and shDHPS #2). β-tubulin is the control for equal protein loading. Empty virus was used as a control. (bottom) Densitometry of p21^CIP1^. (**B**) Time-course trypan blue exclusion assays to determine viability of cells infected as described in (**A**). (**C**) Propidium iodide staining and flowcytometry to determine cell cycle phases of cells treated as described in (**A**). Data are mean ± SD of biological triplicates. * *p* < 0.05, ** *p* < 0.005 and *** *p* < 0.001 by two-tailed Student *t*-test.

Using RNA interference, we determined the effects of DHPS depletion in A375 and SK-MEL-28 that exhibit high MEK/ERK activity due to *BRAF^V600E^* mutation. We found that knockdown of DHPS for 5 days by lentiviruses expressing two independent shRNA, which was sufficient to curtail eIF5A hypusination in cells, consistently increased p21^CIP1^ expression in association with decreased phosphorylation of Rb in these cells (Figure 1A). Rb is a key cell cycle regulator that sequesters the S-phase transcription factor E2F1 [28]. However, DHPS knockdown did not affect the levels of E2F1 and another CDKI p27^KIP1^, while not inducing p16^INK4a^ expression or notably increasing the cleavage of Poly ADP-ribose polymerase (PARP) and lamin A, the apoptotic cell death makers [29,30], in these cells (Figure 1A). Consistent with these changes, DHPS knockdown substantially decreased cell proliferation rates without increasing death rates in these cells (Figure 1B). Moreover, DHPS knockdown increased the G0/G1 phase cell population (Figure 1C). These data demonstrate that DHPS depletion can induce cell cycle arrest which is associated with p21^CIP1^ expression.

### 2.2. eIF5A Depletion Induces Cell Death without Increasing p21^CIP1^ Levels in A375 and SK-MEL-28 Cells

**Figure 2:**
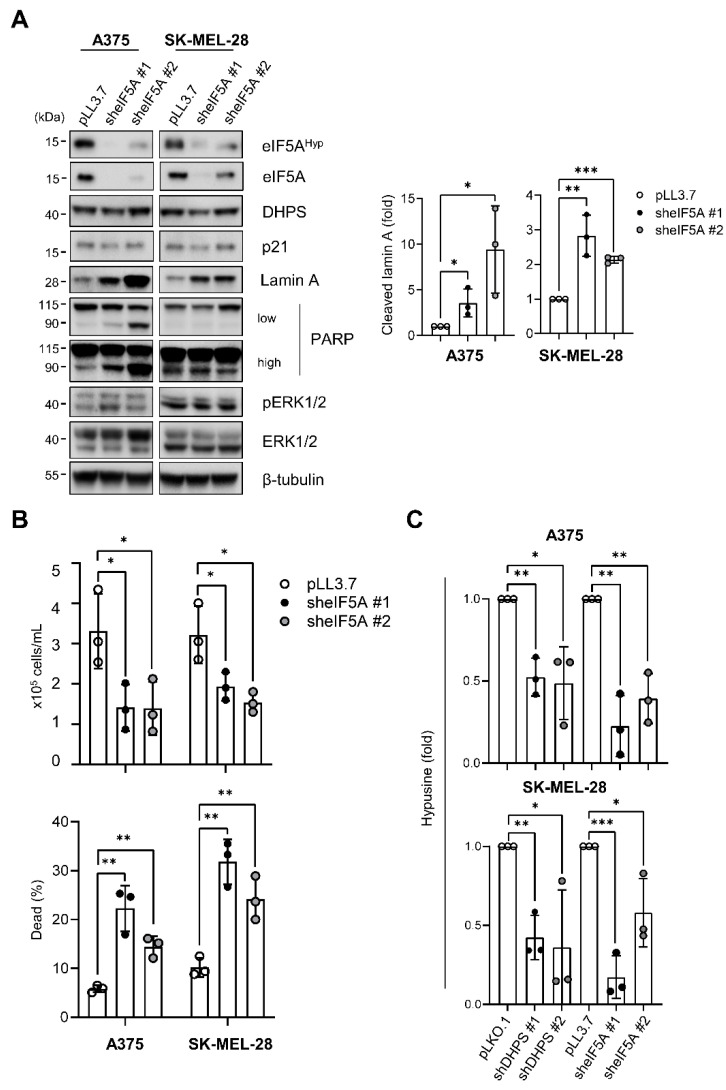
Knockdown of eIF5A induces cell death in A375 andSK-MEL-28 cells. (**A**) Western blots of total lysates of cells infected for 4 days with pLL3.7 viruses expressing shRNA targeting different regions of *EIF5A* mRNA (sheIF5A #1 and sheIF5A #2). β-tubulin is the control for equal protein loading. Empty virus was used as a control. (right) Densitometry of cleaved lamin A normalized to β-tubulin. (**B**) Trypan blue exclusion assays to determine viability of cells infected as described in (**A**). (**C**) Densitometry of hypusine from (Figure 1A) and (Figure 2A) normalized to β-tubulin. Data are mean ± SD of biological triplicates. * *p* < 0.05, ** *p* < 0.005, and *** *p* < 0.001 by two-tailed Student *t*-test.

As determined by Western blotting using an antibody that reacts with both eIF5A1 and eIF5A2 isoforms, RNA interference of eIF5A1 substantially decreased eIF5A levels in SK-MEL-28 and A375 cells (Figure 2A). In these cells, the effects of eIF5A depletion were in stark contrast to the effects of DHPS depletion. eIF5A knockdown in these cells, mediated by two independent lentiviral shRNA constructs, robustly increased the cleavage of lamin A and PARP but did not increase p21^CIP1^ expression (Figure 2A). Consistent with the death markers, eIF5A knockdown substantially increased cell death in the cultures of these cells, as determined by the trypan blue exclusion analysis (Figure 2B). Importantly, DHPS knockdown and eIF5A knockdown decreased eIF5A hypusination at similar degrees (Figure 2C), and did not cause any correlative changes in ERK1/2 phosphorylation in these cells (Figure 1A and Figure 2A). Therefore, the different effects of these treatments are not due to different eIF5A hypusination levels or altered ERK1/2 activity. These data suggested that DHPS and eIF5A may have distinct effects on cell fate and p21^CIP1^ regulation in cells.

The contrasting effects of DHPS and eIF5A knockdowns were not limited to A375 and SK-MEL-28 cells. Similar as these cells, DHPS knockdown suppressed cell proliferation without inducing cell death whereas eIF5A knockdown induced cell death responses, albeit at varied degrees, in the *KRAS*-mutant human pancreatic cell lines, MIA-PaCa-2 and PANC-1 (Supplemental Figure S1A–C). However, unlike in A375 and SK-MEL-28 cells, DHPS depletion did not increase p21^CIP1^ levels in these cells (Supplemental Figure S1A), suggesting that cellular responses to DHPS knockdown are not entirely identical across different cell types.

### 2.3. p21^CIP1^ Depletion Switches DHPS Depletion-Induced Growth Arrest to Cell Death

**Figure 3:**
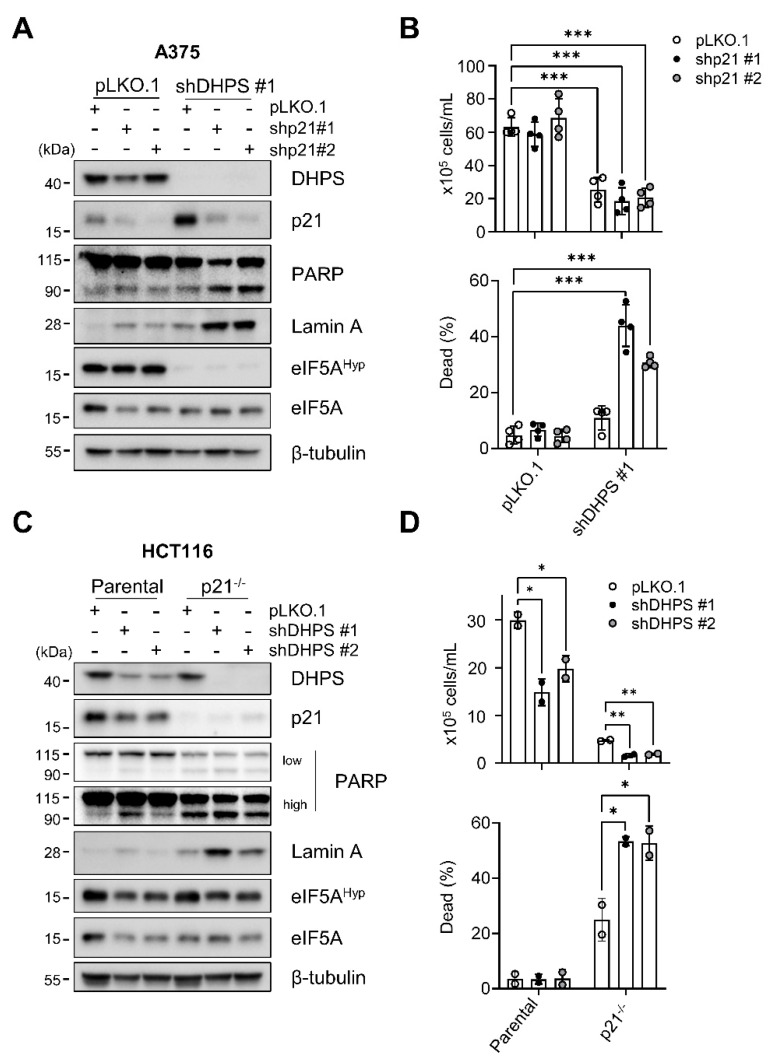
p21^CIP1^ depletion switches DHPS knockdown-induced growth arrest to cell death. (**A**) Western blots of total lysates of cells co-infected for 5 days with pLKO.1-shDHPS virus and pLKO.1 viruses expressing shRNA targeting different regions of *CDKN1A* mRNA (shp21 #1 and shp21 #2). β-tubulin is the control for equal protein loading. Empty virus was used as a control. (**B**) Trypan blue exclusion assays to determine viability of A375 cells infected as described in (**A**). (**C**) Western blots of total lysates of parental and p21^−/−^ HCT116 cells infected with pLKO.1-shDHPS virus for 5 days. (**D**) Trypan blue exclusion assays to determine viability of HCT116 cells infected as described in (**C**). Data are mean ± SD of biological triplicate and duplicate. * *p* < 0.05, ** *p* < 0.005, and *** *p* < 0.001 by two-tailed Student *t*-test.

We previously showed that p21^CIP1^-mediated cell cycle arrest can suppress death responses in MEK/ERK-dependent tumor cells [31,32]. Given this and the data above, we asked whether p21^CIP1^ has a role in determining cell fate in the face of DHPS depletion. Indeed, we found that blocking p21^CIP1^ expression can switch DHPS depletion-induced cell cycle arrest to cell death responses. Knockdown of p21^CIP1^ using lentiviruses expressing two independent shRNA consistently increased the cleavage of PARP and lamin A, and dead cell populations in A375 cultures only when combined with DHPS knockdown (Figure 3A,B). Consistent with these effects, DHPS knockdown increased the cleavage of PARP and lamin A, and induced cell death in CDKN1A (encoding p21^CIP1^)-deleted HCT116 (p21^−/−^) cells, but not in the parental cells (Figure 3C,D). Nevertheless, DHPS knockdown did not increase p21^CIP1^ levels in HCT116 cells, further supporting the notion that DHPS-mediated p21^CIP1^ regulation is cell type specific.

p21^CIP1^ depletion did not alter the effects of DHPS knockdown on eIF5A hypusination in these cells when compared with their parental cells (Figure 3A,C), excluding potential interference by uneven eIF5A hypusination. These data demonstrate that p21^CIP1^ can regulate cellular responses to DHPS depletion.

### 2.4. Hypusinating Activity Is Not Necessary for DHPS to Regulate p21^CIP1^

**Figure 4:**
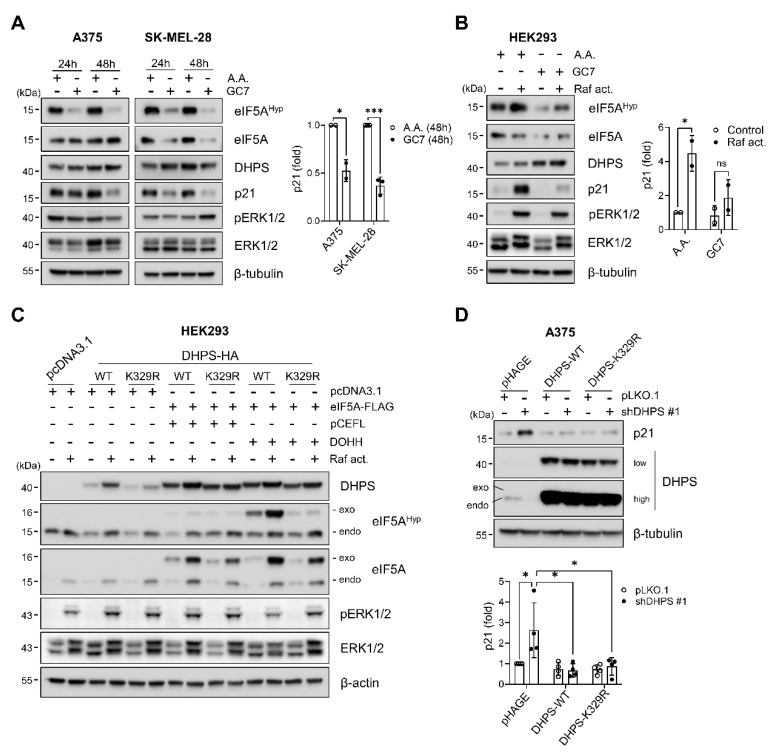
Hypusinating activity is not necessary for DHPS to regulate p21^CIP1^ levels. (**A**) Western blots of total lysates of cells treated with 10 µM GC7 for indicated time periods. Equal volume of acetic acid (A.A.) was used as the vehicle control. (right) Densitometry of p21^CIP1^ normalized to β-tubulin. (**B**) Western blots of total lysates of HEK293-ΔRaf:ER treated with 1 µM tamoxifen (Raf act.) with or without 10 µM GC7 for 24 h. (right) Densitometry of p21^CIP1^ normalized to β-tubulin. (**C**) Western blots of total lysates of HEK293-ΔRaf:ER cells co-transfected for 48 h with pcDNA3.1 plasmids expressing wild type DHPS (WT), DHPS-K329R (K329R), and C-terminal FLAG-tagged eIF5A (eIF5A-FLAG), and pCEFL plasmid expressing DOHH, and then treated with 1 µM tamoxifen (Raf act.) for an additional 24 h. Empty vectors were used as the control. β-actin is the control for equal protein loading. Exo and endo indicate exogenous eIF5A and endogenous eIF5A, respectively. (**D**) Western blots of total lysates of cells co-infected for 5 days with pLKO.1-shDHPS virus and pHAGE viruses expressing DHPS constructs engineered to avoid shDHPS. (bottom) Densitometry of p21^CIP1^ normalized to β-tubulin. Data are mean ± SD of biological duplicate and triplicate. * *p* < 0.05 and *** *p* < 0.001 by two-tailed Student *t*-test.

Because the contrasting knockdown effects of DHPS and eIF5A may suggest an eIF5A-independent role for DHPS, we examined the effects of the spermidine analog GC7 in A375 and SK-MEL-28 cells. GC7 is a competitive inhibitor of DHPS catalytic activity [33], and we expected that GC7 would help us determine whether DHPS knockdown-induced p21^CIP^ was due to depleted enzyme activity or protein. Our time-course analysis revealed that GC7, used at levels sufficient to inhibit eIF5A hypusination, did not increase but decreased the basal levels of p21^CIP1^ in A375 and SK-MEL-28 cells (Figure 4A). As an orthogonal approach, we also examined the effects of GC7 on p21^CIP1^ expression induced upon activation of ΔRaf-1:ER in HEK293 cells. ΔRaf-1:ER is the CR3 catalytic domain of Raf-1 fused to the ligand binding domain of the estrogen receptor for tamoxifen-controlled activity [34]. In these cells, GC7 treatment substantially inhibited ΔRaf-1:ER-induced p21^CIP1^ expression without affecting ERK1/2 phosphorylation (Figure 4B). These data indicated that inhibition of DHPS catalytic activity cannot mimic DHPS depletion in cells, leading us to hypothesize that DHPS has a role independent of its catalytic activity to regulate p21^CIP1^ in cells.

Lys329 of DHPS is necessary for the formation of an enzyme–imine intermediate required for eIF5A hypusination, and replacing this residue with Arg hinders the intermediate formation [35]. To test the aforementioned hypothesis, we generated a DHPS mutant in which Lys329 is replaced with Arg (DHPS-K329R) and determined whether this mutant can regulate p21^CIP1^ levels in cells similarly as wild type DHPS. First, we confirmed that DHPS-K329R is catalytically deficient in HEK293 cells in which DOHH and eIF5A were co-expressed with either DHPS or DHPS-K329R to establish a proper stoichiometry between these proteins. Our data showed that, unlike wild type DHPS, DHPS-K329R cannot increase eIF5A hypusination in this assay (Figure 4C). Expression of this mutant and wild type DHPS did not affect ERK1/2 phosphorylation induced by ΔRaf-1:ER activation (Figure 4C). Next, we determined whether DHPS-K329R, which was engineered to avoid RNA interference, can restore p21^CIP1^ suppression in DHPS-depleted A375 cells. Indeed, when ectopically expressed at similar levels, DHPS-K329R was similarly effective as wild type DHPS for abrogating the effect of DHPS knockdown to increase p21^CIP1^ levels in cells (Figure 4D). These data demonstrate that DHPS can negatively regulate cellular p21^CIP1^ levels independently of its hypusinating activity, thus its substrate eIF5A. We also attempted to determine the ability of DHPS-K329R to rescue cells from DHPS knockdown, but prolonged overexpression of this mutant as well as wild type DHPS suppressed cell proliferation, hampering our efforts. It may be possible that unbalanced stoichiometry between DHPS, DOHH and eIF5A is anti-proliferative.

### 2.5. MEK/ERK Activity Is Necessary for DHPS Knockdown to Increase p21^CIP1^ Levels in Cells

**Figure 5:**
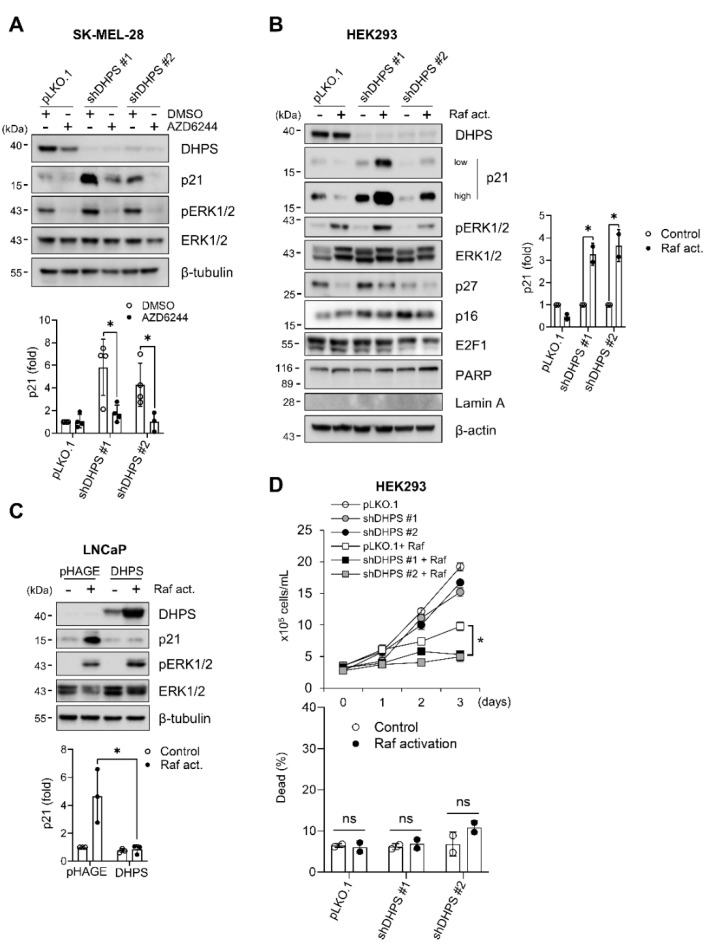
Raf/MEK/ERK activity is necessary for DHPS depletion to increase p21^CIP1^ levels. (**A**) Western blots of total lysates of cells infected with pLKO.1-shDHPS viruses for 4 days, and then treated with 100 nM AZD6244 for an additional 24 h. Equal volume DMSO was used as a vehicle control. (bottom) Densitometry of p21^CIP1^ normalized to β-tubulin. (**B**) Western blots of total lysates of HEK293-ΔRaf:ER cells infected with pLKO.1-shDHPS for 5 days and then treated with 1 µM tamoxifen for an additional 24 h (Raf act.). (right) Densitometry of p21^CIP1^ normalized to β-actin. (**C**) Western blots of total lysates of LNCaP-ΔRaf:ER cells infected with pHAGE-DHPS virus for 48 h and treated with 1 µM tamoxifen for an additional 24 h (Raf act.). (bottom) Densitometry of p21^CIP1^ normalized to β-tubulin. (**D**) Trypan blue exclusion assays to determine viability of HEK293-ΔRaf:ER cells treated as described in (**B**). Cell death was determined at day 3. Data are mean ± SD of biological triplicate and duplicate. * *p* < 0.05 by two-tailed Student *t*-test.

We previously demonstrated that MEK/ERK activity is sufficient to induce p21^CIP1^ [36] and that the MEK/ERK activity in *BRAF*-mutant tumor cells can be routed to induce p21^CIP1^ expression [37]. We therefore determined whether the MEK/ERK activity is necessary for DHPS knockdown to increase p21^CIP1^ levels in cells. First, we found that AZD6244, a highly selective MEK1/2 inhibitor [38], substantially attenuated p21^CIP1^ expression in DHPS depleted SK-MEL-28 cells (Figure 5A). Second, the combination of DHPS knockdown and ΔRaf-1:ER activation robustly increased p21^CIP1^ levels in HEK293 cells without affecting other cell cycle or death effectors (Figure 5B). Conversely, DHPS overexpression in LNCaP cells, a cell line that rapidly expresses p21^CIP1^ in response to ΔRaf-1:ER activation [32,36], substantially abrogated the p21^CIP1^ expression induced by ΔRaf-1:ER activation (Figure 5C). Moreover, consistent with its effect to augment p21^CIP1^ expression, the combination of DHPS knockdown and ΔRaf-1:ER activation more potently suppressed HEK293 cell proliferation than DHPS knockdown or ΔRaf-1:ER activation alone (Figure 5D). However, this combination did not notably induce cell death (Figure 5D), which is consistent with its lack of effects on PARP and lamin A cleavage (Figure 5B). Together, these data suggest that DHPS negatively regulates MEK/ERK-induced p21^CIP1^ expression.

### 2.6. DHPS Knockdown Increases CDKN1A Transcription via TP53 and SP1 in A375 and SK-MEL-28 Cells

**Figure 6:**
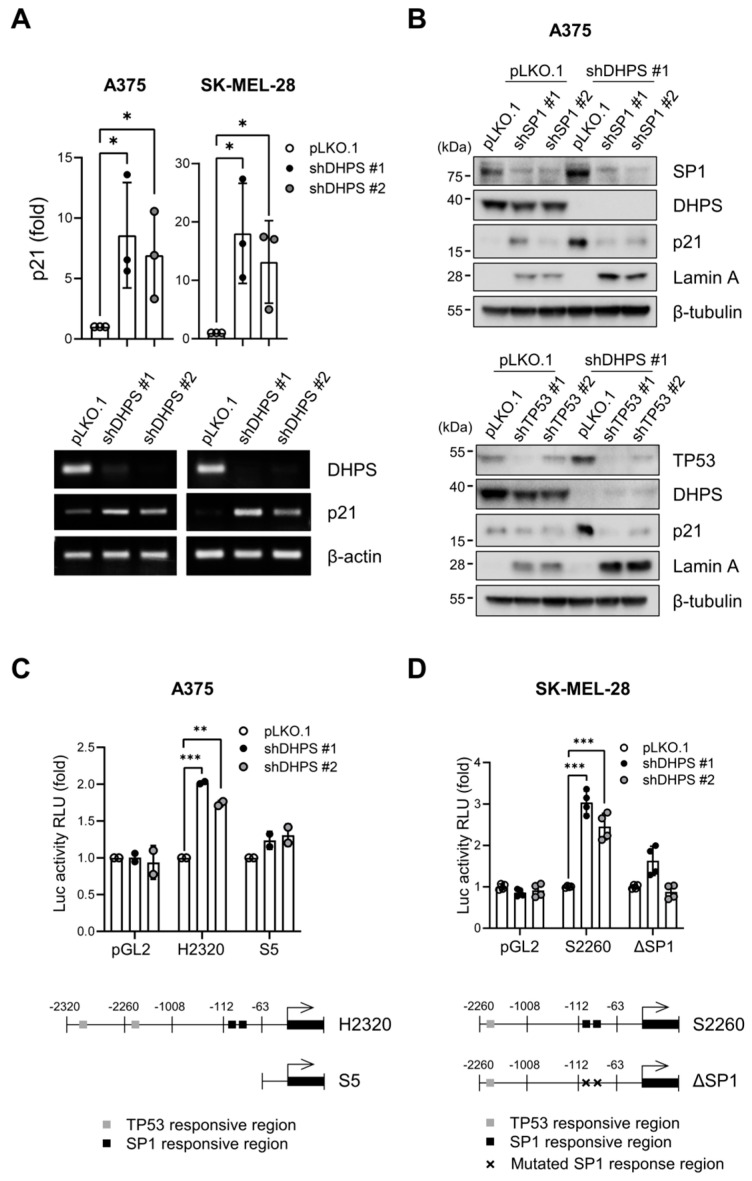
DHPS depletion increases *CDKN1A* transcription via TP53 and SP1 in *BRAF^V600E^* tumor cells. (**A**) (top) qPCR analysis of total RNA of cells infected with pLKO.1-shDHPS for 5 days. (bottom) RT-PCR analysis of the same total RNA samples. β-actin was the control for RNA amounts. (**B**) Western blots of total lysates of cells co-infected for 5 days with pLKO.1-shDHPS virus and pLKO.1-shSP1 viruses expressing shRNA targeting different regions of *SP1* mRNA (shSP1 #1 and shSP1 #2, top panel) or with pLKO.1-shDHPS virus and pLKO.1-TP53 viruses expressing shRNA targeting different regions of *TP53* mRNA (shTP53 #1 and shTP53 #2, bottom panel). (**C**,**D**) Luciferase reporter assays of cells infected with pLKO.1-shDHPS viruses for 5 days. At 2 days post-infection, cells were transfected with the luciferase reporters of p21^CIP1^-promoter, H2320 and S2260, and maintained for 3 days prior to luciferase activity measurement. Data are mean ± SD of biological triplicate and duplicate. * *p* < 0.05, ** *p* < 0.005, and *** *p* < 0.001 by two-tailed Student *t*-test.

p21^CIP1^ is regulated at multiple levels including transcription, translation, and post-translation [39]. We found that DHPS knockdown substantially increased *CDKN1A* (encoding p21^CIP1^) mRNA levels in A375 and SK-MEL-28 cells (Figure 6A), and thus sought to determine the molecular mechanism underlying this increase. Although TP53 is the major regulator of *CDKN1A* transcription [40,41], we previously reported that SP1 can also mediate MEK/ERK-induced *CDKN1A* transcription in tumor cells deficient of a functional TP53 [42]. Accordingly, we examined the effects of TP53 knockdown and SP1 knockdown on DHPS knockdown-induced p21^CIP1^ expression in A375 (*TP53* wild type) and SK-MEL-28 (*TP53^L145R^*) cells. As determined by Western blotting in A375 cells, TP53 knockdown and SP1 knockdown substantially attenuated p21^CIP1^ expression induced by DHPS knockdown while increasing lamin-A cleavage (Figure 6B). This effect is consistent with p21^CIP1^ depletion effects in the background of DHPS knockdown. Moreover, DHPS knockdown increased the activity of the luciferase reporter, H2320, that contains 2320 base pairs of *CDKN1A* promoter in A375 cells, but this increase was abrogated upon truncation of the region that contain TP53 and SP1 responsive elements (Figure 6C). Similarly, DHPS knockdown upregulated the activity of the luciferase reporter, S2260, that contains 2260 base pairs of *CDKN1A* promoter in SK-MEL-28 cells but this induction was abrogated upon disabling the SP1 binding sites by site-directed mutagenesis (Figure 6D). These data suggest that DHPS knockdown increases p21^CIP1^ expression by promoting *CDKN1A* transcription through TP53 or SP1 in these cells.

## 3. Discussion

**Figure 7:**
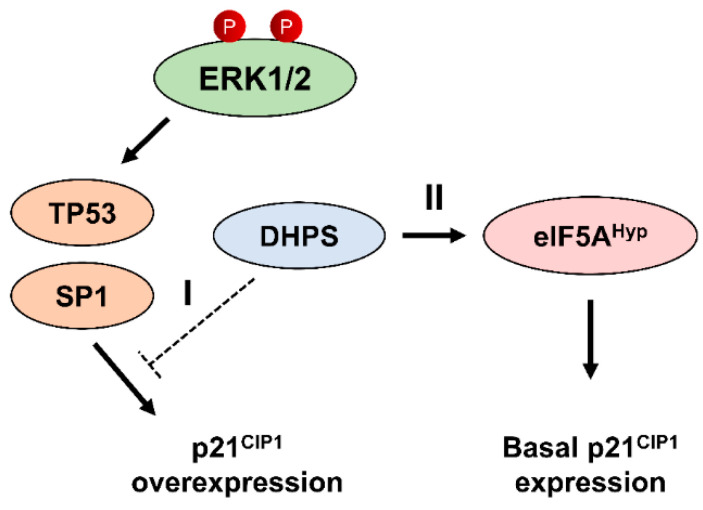
A proposed role for DHPS in p21^CIP1^ regulation in cells. (I) DHPS suppresses p21^CIP1^ overexpression, which can be induced by aberrant ERK1/2 activity and mediated by TP53 and SP1 in certain tumor cells. This DHPS function is independent of its ability to hypusinate eIF5A. (II) DHPS can also regulate basal p21^CIP1^ levels in cells through eIF5A hypusination. These mechnanisms may be involved in p21^CIP1^-mediated regulation of cell proliferation and survival.

In this report, we demonstrate that, although known for their exclusive enzyme–substrate relationship and thus expected to mediate largely overlapping cellular processes [5], DHPS and eIF5A depletion can result in distinct effects on cell fate. Mechanistically, our data suggest that DHPS has a novel eIF5A hypusination-independent function that can negatively regulate p21^CIP1^ expression in cells by suppressing TP53- and SP1-mediated MEK/ERK activity-sensitive *CDKN1A* transcription (a model depicted in Figure 7). Given that DHPS does not interact with TP53 and SP1 (data not shown), we speculate that DHPS indirectly regulates *CDKN1A* transcription in this model, although the detailed molecular mechanism requires to be elucidated.

Our data provide compelling evidence supporting that DHPS can regulate cell fate independently of its activity to catalyze hypusination, thus of its only known substrate eIF5A. First, knockdown of DHPS and eIF5A produced different physiological effects in A375, SK-MEL-28, MIA-PaCa-2, PANC-1, and HCT116 cell lines. Whereas DHPS knockdown induced growth arrest, direct knockdown of eIF5A induced cell death accompanied by increased cleavage of lamin A and PARP, albeit at varied levels. In these cells, DHPS knockdown and eIF5A knockdown reduced eIF5A hypusination to similar degrees, and knockdown of one did not affect the expression of the other. Therefore, we suggest that the differential outcomes in cell fate were not a result of varying levels of eIF5A hypusination but probably a specific effect due to distinct roles of DHPS and eIF5A. Second, our data suggest a clear functional difference between DHPS and eIF5A in p21^CIP1^ regulation. Whereas DHPS knockdown increased p21^CIP1^ expression in A375 and SK-MEL-28 cells, eIF5A knockdown decreased it. Third, similar as eIF5A knockdown, GC7 decreased basal p21^CIP1^ expression in these cells while exhibiting higher potency than eIF5A knockdown, which suggested that protein depletion but not hypusinating activity underlies the p21^CIP1^-inducing effect of DHPS knockdown. This notion is strongly supported by the data obtained with the catalytically disabled DHPS-K329R mutant. Given these contrasting effects on p21^CIP1^, we suspect that while DHPS maintains basal p21^CIP1^ expression via an eIF5A-dependent mechanism, which fits into the context of the global effect of hypusinated eIF5A to promote protein translation [16], DHPS also regulates an eIF5A-independent mechanism to prevent p21^CIP1^ overexpression caused by an abnormal signal such as hyper ERK1/2 activity (Figure 7).

Aberrant MEK/ERK activity can cause cell cycle arrest for which p21^CIP1^ induction is a key mechanism [32,36,37]. This anti-proliferative response addresses in part oncogene-induced senescence, a hypothetical innate tumor defense mechanism in cells [43]. Our data suggest a role for DHPS in this context, given that the aberrant MEK/ERK activity was necessary for DHPS knockdown to induce p21^CIP1^ expression in A375 and SK-MEL-28 cells, and ΔRaf-1:ER-activated HEK293 cells. Because DHPS knockdown or overexpression did not affect ERK1/2 phosphorylation in these cells, DHPS might function at a level downstream of ERK1/2. Indeed, our data suggest that DHPS regulates MEK/ERK-induced p21^CIP1^ expression through the transcription factors TP53 and SP1. While TP53 is a bonafide regulator for *CDKN1A* transcription [40], we previously reported that SP1 also facilitates *CDKN1A* transcription in response to aberrant MEK/ERK activity [42]. Interestingly, despite the presence of intact TP53, SP1 knockdown substantially abrogated p21^CIP1^ expression induced by DHPS knockdown in A375 cells. Of note, it was reported that TP53 can recruit SP1 to p21^CIP1^ promoter and this enhances the binding of these transcription factors to the DNA promoter [44]. Given this information, it may be possible that DHPS has a role in promoting the cooperative effects of SP1 and TP53. How DHPS functionally interacts with TP53 and SP1 remains to be elucidated in future work.

In summary, we report a previously unknown non-canonical DHPS function, which is independent of its activity to hypusinate eIF5A and regulates p21^CIP1^ expression induced by MEK/ERK via TP53 and SP1. We propose that this function may be important for *BRAF*-mutated tumor cells to suppress p21^CIP1^ overexpression, which can be triggered by aberrant MEK/ERK activity in these tumor cells if not prevented.

## 4. Materials and Methods

### 4.1. Cell Culture and Reagents

The human embryonic kidney cell line HEK293 (ATCC, Manassas, VA, USA) was maintained in minimal essential medium (Invitrogen, Waltham, MA, USA, 11095-080) supplemented with 10% fetal bovine serum (Gibco, Waltham, MA, USA, SH30541.03). The human prostate cancer cell line LNCaP (ATCC, Manassas, VA, USA) was maintained in phenol-red free RPMI (Invitrogen, Waltham, MA, USA, 11835-030) supplemented with 10% fetal bovine serum. The human melanoma cell line SK-MEL-28 (ATCC, Manassas, VA) were maintained in minimal essential medium supplemented with 10% fetal bovine serum, 100U/mL penicillin, and 100 μg/mL streptomycin (Gibco, Waltham, MA, USA, 15140-122), 1% sodium pyruvate (Gibco, Waltham, MA, USA, 11360-070), and 1% non-essential amino acids (Gibco, Waltham, MA, USA, 11140-050). The human melanoma cell line A375 (ATCC, Manassas, VA, USA), and human pancreatic cancer cell lines MIA-PaCa-2 and PANC-1 were maintained in Dulbecco’s modified eagle medium (Invitrogen, Waltham, MA, USA, 11965-092) with 10% fetal bovine serum, 100U/mL penicillin, and 100 μg/mL streptomycin. The human colorectal carcinoma cell line HCT116 was maintained in McCoy’s 5a medium (Gibco, Waltham, MA, USA, 16600) with 10% fetal bovine serum, 100U/mL penicillin and 100 μg/mL streptomycin. Generation of LNCaP-ΔRaf:ER and HEK293-ΔRaf:ER cells that are stably transduced with the lentiviral pHAGE-ΔRaf:ER was previously described [32,36]. ΔRaf:ER was activated by treating cells with 1 µM 4-Hydroxytamoxifen (Sigma, St. Louis, MO, USA, 68392-35-8) as previously described [34]. AZD6244 (606143-52-6) and PLX4032 (S1267) were obtained from Selleck Chemicals.

### 4.2. Plasmids and Viral Constructs for Gene Expression and RNA Interference

C-terminally HA-tagged DHPS in pHAGE-GFP was generated by ligating the full length DHPS cDNA from pCMV3-ORF-HA (Sino Biologicals, Bejing China, HG14407-CY) into the NheI/XhoI sites of pHAGE-GFP. pcDNA3.1-eIF5A and pCEFL-DOHH were obtained from Dr. Myung Hee Park [11,13]. DHPS was depleted using two independent pLKO.1 lentiviral shRNA systems (Sigma-Aldrich, St. Louis, MO, USA, TRCN0000045644 labeled as shDHPS-#1 and TRCN00000330717 labeled as shDHPS-#2). pLKO.1-shDHPS-#1 and pLKO.1-shDHPS-#2 target GCGACATGATCTTCTTCCATTC and AGTGCACTGGGATGATCATTC, respectively. Lentiviral shRNA expression systems pLL3.7-sheIF5A-#1 and pLL3.7-sheIF5A-#2 target GCATTACGTAAGAATGGCTTT and CTGGGAAGAAATATGAAGATA of *EIF5A* mRNA, respectively. pLL3.7-sheIF5A-#1 was generated by ligating the annealing product of TGCATTACGTAAGAATGGCTTTCTCGAGAAAGCCATTCTTACGTAATGCT TTTTC and TCGAGAAAAAGCATTACGTAAGAATGGCTTTCTCGAGAAAGCCATTCTT ACGTAATGCA into the XhoI/HpaII sites of pLL3.7 (ATCC). pLL3.7-sheIF5A-#2 was generated by ligating the annealing product of TCTGGGAAGAAATATGAAGATACTCGA GTATCTTCATATTTCTTCCCA GTTTTTC and TCGAGAAAAACTGGGAAGAAATATGA AGATACTCGAGTATCTTCATATTTCTTCCCAGA into the XhoI/HpaII sites of pLL3.7. To generate DHPS-K329R, the wild type DHPS in pHAGE was mutagenized using the QuickChange Lightning Site-Directed Mutagenesis Kit (Agilent Technologies, Santa Clara, CA, USA, 210518-5) and the primers GGCTGTCTCCTGGGGCAGGATCCGGGTGGAT and GCATCCACCCG GATCCTGCCCCAGGAGACAG. To generate shRNA non-targetable constructs, pHAGE-DHPS and pHAGE-DHPS-K329R were mutagenized using the primers GCTCGCTGGGC GACATGATTTTTTTTCATTCCTACAAGAACC and GGTTCTTGTAGGAATGAAAAAAAA TCATGTCGCCCAGCGAGC p21^CIP1^ was depleted using two independent pLKO.1 lentiviral shRNA systems (Sigma-Aldrich, St. Louis, MO, USA, TRCN0000040123 labeled as shp21 #1 and TRCN0000040125 labeled as shp21 #2). pLKO.1-shp21 #1 and pLKO.1-shp21 #2 target CGCTCTACATCTTCTGCCTTA and GAGCGATGGAACTTCGACTTT, respectively.

For lentivirus production, 293T cells were co-transfected with the lentiviral backbone and packaging vectors, as previously described [36]. Viral supernatants were collected after 72 h. Viral titers were determined by infecting the recipient cell lines with serially diluted viral supernatants, and then scoring cells expressing GFP at 48 h post-infection.

### 4.3. Analysis of Cell Viability and Cell Cycle

Cell viability was measured by trypan blue exclusion assay. The cell cycle analysis was performed as previously described [36]. Briefly, cells were washed with ice-cold 0.2% bovine serum albumin in phosphate-buffer saline, and then resuspended in 250 mM sucrose/40mM citrate buffer (pH 7.6) containing 0.5% dimethylsulfoxide. Cell nuclei were stained with propidium iodide and analyzed using the Guava EasyCyte flowcytometry system (Millipore Sigma, St. Louis, MO, USA). Gating targeted single cell nuclei within a normal size range. The cell-cycle parameters were determined from 5000 gated nuclei, and then analyzed with FCS EXPRESS 6 FLOW software (De Novo Software, Boulder, CO, USA).

### 4.4. Quantitative RT-PCR (qPCR) and Luciferase Reporter Assays

TRIzol^®^ reagent (Invitrogen, Waltham, MA, USA, 15596026) was used to isolate total RNA from cells. Reverse transcription was performed using Superscript III (Invitrogen, Waltham, MA, USA, 18080-044) and oligo-dT according to the manufacturer’s instructions. Quantitative RT-PCR was performed by mixing the resulting cDNA with the SYBR GreenER qPCR Supermix Universal (Invitrogen, Waltham, MA, USA, 11762100) and the following primers: CTGGAGACTCTCAGGGTCGAA and CCAGCACTCTT AGGAACCTCTCA for p21^CIP1^; AAGTTTGAGGACTGGCTGATG and CAGGGATGTGGT TCTTCTGG for DHPS; and GTCCTCTCCCAAGTCCACAC and GGGAGA CCAAAAGCCTTC AT for β-actin. qPCR signal was obtained using a Stratagene MX3005P instrument.

Construction of the p21^CIP1^ promoter luciferase reporters H2320, S2260, and S5 that contains p21^CIP1^ promoter DNA fragments spanning −2320 to +16, −2260 to +16, and −63 to +16 base pairs respectively, were previously described [42]. To generate ΔSP1, two SP1 sites in S2260 were mutated using the QuikChange Lightning site-directed mutagenesis kit (Agilent Technologies, Santa Clara, CA, USA 210518-5) and the primers GTGGGCCGAGCGCGGGTCTTACCTCCTTGAGGTAAGCC and GGCTTACCTCAAGG AGGTAAGACCCGCGCTCGGCCCAC. To measure the reporter activity, A375 and SK-MEL-28 cells were transfected in 6-well plates using Lipofectamine 3000 (Invitrogen, Waltham MA, USA, L3000-008) or Lipofectamine LTX (Invitrogen, Waltham, MA, USA, 15338-100) respectively, and total cell lysates were extracted and analyzed using the Luciferase^®^ Assay System (Promega, Madison, WI, USA, E1960) according to the manufacturer’s instructions. Data were normalized to total protein concentrations.

### 4.5. Immunoblotting

Cells were harvested in lysis buffer containing 62.5 mM Tris-HCl (pH 6.8)/2% SDS, and protease and phosphatase inhibitor cocktails 2 and 3 (Sigma-Aldrich, St. Louis, MO, USA, P8340, P5726, P0044). Protein concentrations were measured using the bicinchoninic acid reagent (Pierce, Waltham, MA, USA, 23228, 1859078). Proteins were resolved by sodium dodecyl sulfate polyacrylamide gel electrophoresis and transferred to polyvinylidene difluoride membrane filter (Bio-Rad, Hercules, CA, USA, 1620177). After transfer, membranes were blocked at 25 °C for 1h in buffer containing 0.1M Tris (pH 7.4), 0.9% NaCl, 0.05% Tween 20, and 5% nonfat dry milk.

Membranes were then incubated with the appropriate antibodies overnight at 4 °C at the dilutions indicated as follows: phospho-ERK1/2 1:2000 (Thr202/Tyr204, sc-16982), DHPS 1:2000 (sc-365077), eIF5A 1:2000 (sc-390202), p21^CIP1^ 1:1000 (sc-756), p27 1:1000 (sc-528), E2F1 1:1000 (sc-193), p16 1:1000 (sc-1661) (Santa Cruz Biotechnology, Santa Cruz, CA, USA). PARP (9542S), Lamin A (2035S), ERK1/2 1:2000 (9102S), pRb 1:2000 (S780, 9307S), Rb 1:2000 (9309P), (Cell Signaling Technology, Danvers, MA, USA). β-actin 1:5000 (Sigma, St. Louis MO, USA, A1978), β-tubulin 1:5000 (Invitrogen, Waltham MA, USA, PA5-16863), eIF5A^Hyp^ 1:5000 (gift from Dr. Mirmira [45]). SuperSignal West Pico and Femto chemiluminescence kit (Pierce, Waltham, MA, USA, 34094) were used for visualization of the signal. For densitometry, immunoblots were analyzed using Image Lab software (Bio-Rad, Hercules, CA, USA).

### 4.6. Statistical Analysis

Statistical significance was determined using the two-tailed unpaired Student’s *t*-test using PRISM (Graph-Pad Software, La Jolla, CA, USA) of two data sets. *p* values of <0.05 were considered statistically significant.

## Data Availability

Not applicable.

## Supplementary Materials

The following are available online at https://www.mdpi.com/article/10.3390/ijms222413187/s1.
