# Supplementary material for: eIF5A-Independent Role of DHPS in p21CIP1 and Cell Fate Regulation
Source: Int J Mol Sci. 2021 Dec 7;22(24):13187. doi: 10.3390/ijms222413187 (PMC8707118; doi:10.3390/ijms222413187)
Supplement: Supplementary file 1 [file ijms-22-13187-s001.zip › ijms-1471377-supplementary.pdf]

Supplemental Materials:

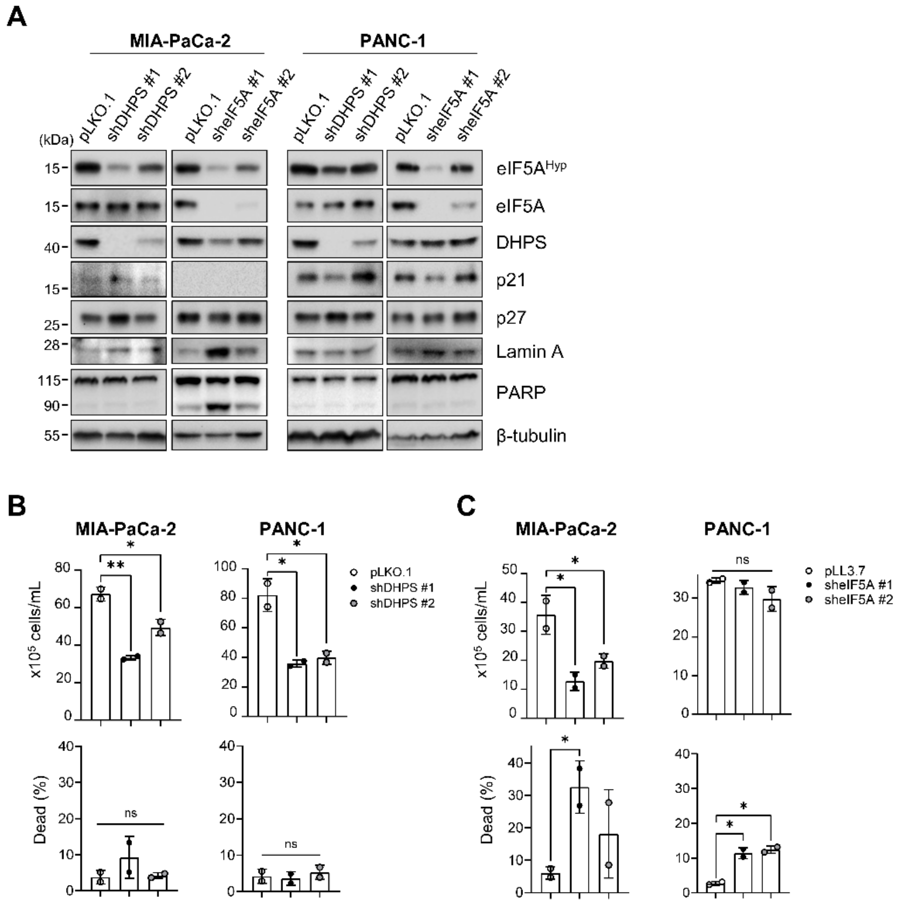

Figure S1 (A) Western blots of total lysates of cells infected for 5 days with pLKO.1 viruses expressing shRNA targeting different regions of *DHPS* mRNA or *EIF5A* mRNA. β-tubulin is the control for equal protein loading. Empty virus was used as a control. **(B)** Trypan blue exclusion assays to determine viability of cells infected with shDHPS viruses for 5 days. **(C)** Trypan blue exclusion assays to determine viability of cells infected with sheIF5A viruses for 5 days. Data are mean ± SD of biological duplicate. * $P < 0.05$ and ** $P < 0.005$ by two-tailed Student t-test.
